# Examining drug poisoning in child and adolescent psychiatric patients: clinical analysis and pharmacy services

**DOI:** 10.3389/fpsyt.2025.1507639

**Published:** 2025-07-11

**Authors:** Yuwen Wang, Yuejing Wu, Rongda Wang, Zemin Wang, Fugang Luo

**Affiliations:** ^1^ Department of Pharmacy, Affiliated Mental Health Center & Hangzhou Seventh People’s Hospital, Zhejiang University School of Medicine, Hangzhou, China; ^2^ Department of Critical Care Medicine, Affiliated Mental Health Center & Hangzhou Seventh People’s Hospital, Zhejiang University School of Medicine, Hangzhou, China

**Keywords:** adolescent, drug poisoning, psychiatric conditions, drug overdose, pharmaceutical services, psychiatric medications

## Abstract

**Objectives:**

This study aimed to investigate the clinical characteristics and symptoms of drug poisoning in adolescents with psychiatric conditions, and provide valuable guidance for pharmacists in the prevention and treatment of adolescent drug poisoning.

**Methods:**

A total of 84 adolescent patients with drug poisoning were recruited from January 2021 to December 2023. Clinical data of drug poisoning patients were analyzed, and pharmaceutical service implications were discussed based on hospital settings and patient medication profiles.

**Results:**

Female adolescents constituted a higher proportion of drug poisoning cases than males. Over 50% cases involved the overdose of two or more drugs, with quantities ranging from several pills to over a hundred. The drugs most commonly involved in overdose were psychiatric medications, including antidepressants (50.0%), antipsychotics (41.7%), sedatives/hypnotics (35.7%), and mood stabilizers (26.2%). Poisoning symptoms predominantly affected the nervous system, such as dizziness, headache, drowsiness, and unsteady gait, with most patients showing improvement after symptomatic treatment (94.0%).

**Conclusion:**

Our study highlights the complex relationship between psychiatric disorders and intentional drug overdose among adolescents. Effective prevention strategies, including education on poison prevention, safe medication storage, and safety planning, are crucial for reducing intentional drug overdoses in this vulnerable population.

## 1 Introduction

Mental health problems among children and adolescents represent a significant global public health concern, profoundly impacting individuals, families, and societies. According to the World Health Organization (WHO), approximately one in seven individuals aged 10-19 worldwide suffers from mental disorders, contributing to 13% of the global disease burden in this age group. The Centers for Disease Control and Prevention (CDC) also report an increase in the prevalence of mental health disorders among adolescents. For instance, a study by Johnston et al. (1), using data from the National Survey on Drug Use and Health, found that major depressive episodes among adolescents aged 12 to 17 rose from 8.7% in 2005 to 13.2% in 2017. Similarly, the prevalence of diagnosed anxiety disorders in children and adolescents aged 6 to 17 increased from 5.5% in 2003 to 6.4% in 2011-2012, as reported by the CDC (2). Recent studies estimate that 10-20% of children and adolescents worldwide experience mental health disorders, yet only approximately 5% receive any form of mental health care. Suicide is the second leading cause of death among young people, with approximately 52,000 deaths annually in those aged 5-19 years (3). A previous study has indicated a significant concern regarding medication overdoses, whether intentional or unintentional, among psychiatric adolescents. Bond et al. (4) analyzed data from emergency department visits, revealing a rising number of medication overdose cases among adolescents over a ten-year period. Intentional overdose is a common method of suicide attempt, influenced by various social and psychological factors. Recent data highlight rising suicide rates in some regions, particularly among young females in the UK, where rates have increased by 8.5% annually since 2012 (5). The COVID-19 pandemic has further exacerbated adolescent mental health challenges, leading to increased demand for treatment, including psychotropic medications (3).

Adolescents prescribed psychiatric medications, such as antidepressants, antipsychotics, and sedatives/hypnotics, are at increased risk of medication overdose. Several studies have highlighted the association between psychiatric medication use and overdose risk among adolescents. For instance, Hammand et al. (6) examined the relationship between antidepressant use and suicide risk in adolescents, emphasizing the importance of close monitoring and careful prescribing practices. Various risk factors contribute to medication overdose among adolescents. These include demographic factors such as being female, experiencing domestic violence, and a history of previous overdoses (7). Additionally, psychiatric comorbidities such as depression, impulsivity, and substance abuse increase the likelihood of medication overdose (7). The rising prevalence of psychological disorders among adolescents has led to an increased use of psychotropic medications, some of which have been implicated in suicide attempts. Between 2013 and 2022, psychotropic drug prescriptions in this population grew significantly, with fluctuating antidepressant use, stable stimulant prescriptions, and a decline in antipsychotic prescribing. A systematic review by Altuwairqi (8) further highlights an increase in the use of stimulants, antipsychotics, antidepressants, and mood stabilizers for treating psychiatric disorders in young populations. Similarly, a longitudinal study by Brauer et al. (9) analyzing psychotropic medicine consumption in 65 countries from 2008 to 2019 highlights global prescription trends, suggesting a sustained rise in usage even before the pandemic.

Drug overdose symptoms may vary depending on the type of medication but commonly include central nervous system depression, cardiovascular abnormalities, and gastrointestinal disturbances. For instance, antidepressant overdoses, particularly tricyclic antidepressants, are associated with cardiac toxicity, seizures, and respiratory depression (10). Similarly, benzodiazepine overdoses typically result in sedation, respiratory depression, and, in severe cases, coma (11). Stimulant overdoses, such as those involving amphetamines, may present with hypertension, tachycardia, and hyperthermia (12). Given the varying overdose effects across psychotropic drug classes, it is crucial to recognize and manage these symptoms promptly (13, 14). Several approaches have been proposed to prevent medication overdose among adolescents. These include educating patients and caregivers about safe medication use, promoting proper medication storage and disposal practices, implementing screening tools to identify individuals at risk of overdose, and providing access to mental health resources and support services (15). Interventions such as counseling, psychoeducation, and medication management programs have shown promise in reducing medication misuse and overdose risk among adolescents (16). Clinical pharmacists are essential in managing pediatric psychiatric medications, ensuring safety, optimizing therapy, and preventing overdose. They conduct medication reviews, identify drug-induced conditions, and educate patients and caregivers to improve adherence and reduce adverse effects. Pharmacist-led interventions have been shown to enhance treatment outcomes and mitigate overdose risks (17). National healthcare policies shape psychiatric medication use by influencing prescribing patterns, clinician roles, and overdose prevention strategies. In some systems, lenient insurance policies facilitate off-label psychotropic medication use in children and adolescents, contributing to increased prescriptions (17).

The present study examines 84 cases of acute drug poisoning in adolescents treated at our Intensive Care Medicine Department from January 2021 to December 2023. The study aims to investigate the clinical characteristics and symptoms of drug poisoning in adolescents with psychiatric conditions, and provide valuable guidance for pharmacists in the prevention and treatment of adolescent drug poisoning, contributing to treatment, education, and prevention efforts targeting drug poisoning among minors.

## 2 Materials and methods

### 2.1 General information

The Zhejiang University School of Medicine Affiliated Mental Health Center is a top-tier (Grade III, Class A) specialized hospital for mental health care, located in Hangzhou, Zhejiang Province. As one of the largest psychiatric centers in eastern China, it delivers specialized psychiatric care to both urban and rural populations, and regularly receives patients from across the province and neighboring regions. National surveillance data indicate that the prevalence of mental illness, patterns of drug prescription, and rates of suicide attempts at this facility are consistent with national averages. This enhances the relevance of the study findings to other regions in China and potentially to comparable settings internationally.

Between January 2021 and December 2023, 84 pediatric and adolescent cases of drug poisoning were admitted to the Intensive Care Medicine Department. Inclusion criteria were as follows: 1) Individuals under the age of 18; and 2) Cases of drug poisoning diagnosed by clinical assessment and toxicology screening. Exclusion criteria were: 1) Patients readmitted for the same poisoning event to avoid data duplication; 2) Patients with unrelated comorbid conditions; and 3) Cases involving non-pharmaceutical poisoning (e.g., pesticides, toxic gases, recreational drugs, or alcohols). Ethical approval for this research was obtained from the Ethics Committee of Hangzhou Seventh People’s Hospital (Approval No. 2024030).

### 2.2 Data acquisition

This retrospective analysis included 84 cases of drug poisoning in children and adolescents treated at our hospital between 2021 and 2023. Data were extracted from patients’ medical records using a structured abstraction form developed by the research team to ensure consistency across cases. Key data points included age, gender, psychiatric and medical history, clinical diagnosis, substances involved, symptoms, interventions, and clinical outcomes. The questionnaire was completed by clinicians during hospitalization and updated as needed through follow-up interviews with patients and/or caregivers. Psychiatric evaluations were conducted by attending psychiatrists once the patient was clinically stable, including those admitted to the intensive care unit (ICU), which specializes in the care of critically ill psychiatric patients. Clinical pharmacists contributed to medication history review, poisoning analysis, and pharmacological management recommendations.

### 2.3 Statistical analysis

Following data collection, demographic information and clinical parameters were subjected to statistical analysis using SPSS 25.0 software. Descriptive statistics were employed for categorical data, presenting percentages to illustrate the distribution of various factors within the dataset. Continuous data, such as patient age and clinical parameters, were expressed as means ± standard deviations.

## 3 Results

### 3.1 Baseline patient information

Among the 84 cases of adolescent drug poisoning, 14 were male (16.7%) and 70 were female (83.3%), with a mean age of 14.7 ± 1.6 years. Fifty-four cases resided in the local area, 21 cases were from other regions within the province, and 9 cases were from outside the province. Only 2 of the 84 patients had no prior history of psychiatric treatment; however, these 2 patients had severe physical illnesses. Diagnostic findings revealed a predominance of depressive disorders, affecting 53.6% of the cohort. Mood disorders were also prevalent, affecting 33.3% of cases, while childhood emotional disorders accounted for 9.5% of diagnoses. Additionally, the assessment of suicide risk revealed that the majority of patients (70.2%) were classified as having a moderate risk of suicide, while a minority of patients (4.8%) were classified as being at high risk of suicide. Interestingly, the vast majority of patients (94.0%) demonstrated improvement after receiving symptomatic treatment (**Table 1**).

**Table 1:** Demographic and clinical characteristics.

Characteristics	Mean ± SD or n (%)
Age (years)	14.7 ± 1.6
Gender	
Male	14 (16.7)
Female	70 (83.3)
Duration of symptoms	
≤1 year	37 (44.0)
1-3 years	39 (46.4)
>3 years	8 (9.5)
Residential location	
Local area	54 (64.3)
Within the province	21 (25.0)
Outside the province	9 (10.7)
Psychiatric diagnosis	
Depressive disorders	45 (53.6)
Mood disorders	28 (33.3)
Childhood emotional disorders	8 (9.5)
Schizophrenia	1 (1.2)
No psychiatric diagnosis	2 (2.4)
Suicide risk assessment	
Scores 30-41	4 (4.8)
Scores 20-29	59 (70.2)
Scores 10-19	21 (25.0)
Scores <10	0
Outcome	
Recovered	79 (94.0)
Unchanged	2 (2.4)
Unresolved	3 (3.6)

A significant gender disparity was observed, with females comprising the majority of cases at 83.3%, compared to males at 16.7%. Patients affected by drug poisoning in the study ranged in age from 11 to 18 years old. In addition, the peak incidence of drug poisoning showed a slight difference between genders, with females experiencing the highest incidence around the age of 15 and males around the age of 16. None of the 84 patients included in this study reported the use of substances that could enhance the efficacy of the medications they were taking, such as alcohol or other psychoactive substances. All patients were only using the prescribed medications or, in a few cases, medications obtained through other means such as over-the-counter (OTC) drugs or family prescriptions.

### 3.2 Types of drug poisoning

The analysis of drug involvement in overdose cases revealed the prevalent types of drugs (e.g., antidepressants, antipsychotics, sedatives, and mood stabilizers) and their frequency in such incidents (**Table 2**). Among these, antidepressants, including sertraline, fluoxetine, fluvoxamine, venlafaxine, and mianserin, were implicated in a significant portion of the overdose incidents, accounting for 50.0% of cases. Following closely behind antidepressants, antipsychotics, such as quetiapine, aripiprazole, olanzapine, risperidone, and others, contributed to 41.7% of overdose cases. Sedatives and hypnotics, essential for managing anxiety and sleep disorders, were also implicated in a substantial proportion (35.7%) of overdose cases. Drugs such as lorazepam, oxazepam, zopiclone, alprazolam, and others were involved. Mood stabilizers and anticonvulsants, crucial for managing bipolar disorder and seizures, were identified in 26.2% of cases. Lithium carbonate, sodium valproate, and lamotrigine were among the drugs implicated. Furthermore, the analysis revealed instances of patients overdosing on a combination of drugs, with some cases involving the ingestion of multiple substances. Notably, 40 of the cases involved a single drug overdose, 18 involved the ingestion of two drugs concurrently, and 25 involved three or more drugs. However, one patient’s drug usage remained unspecified.

**Table 2:** Types of toxic drugs.

Drug type	Representative drugs (instances)	Cases/n (%)
Antidepressants	Sertraline (30), fluoxetine (7), fluvoxamine (10), venlafaxine (1), mianserin (1)	42 (50.0%)
Antipsychotics	Quetiapine (22), aripiprazole (5), olanzapine (4), risperidone (2), chlorpromazine (1), hydroxyrisperidone (1), lurasidone (1), promethazine (1)	35 (41.7%)
Sedatives/Hypnotics	Lorazepam (19), oxazepam (3), zopiclone (3), alprazolam (2), clonazepam (1), diazepam (1), eszopiclone (1)	30 (35.7%)
Mood stabilizers/Anticonvulsant	Lithium carbonate (16), sodium valproate (8), lamotrigine (2), levetiracetam (1), topiramate (1), pregabalin (1)	22 (26.2%)
Other nervous system drugs	Phenobarbital (3), traditional chinese medicine (3), mefenamic acid (2), metadoxine (1), fluphenazine (1), amantadine (1), ropinirole (1)	11 (13.1%)
Non-neurological drugs	Cephalosporin antibiotics (3), antiallergic drugs (1), montelukast (1), etofenamate (1)	6 (7.1%)

### 3.3 Symptoms and outcome of drug poisoning

As shown in **Table 3**, various symptoms such as neurological, gastrointestinal, psychiatric, cardiovascular, respiratory, urinary, and other systems were reported among affected patients. Neurological symptoms predominated among the clinical presentations, with dizziness being the most commonly reported symptom, affecting over half of the cases (51.2%). Additionally, patients frequently exhibited symptoms such as unsteady gait (29.8%), drowsiness (25.0%), tremor (17.8%), and slurred speech (17.8%), indicating the profound impact of drug toxicity on neurologic function. Other neurological manifestations included confusion, headache, convulsions, muscle stiffness, hyperactivity, facial asymmetry, delirium, tinnitus, and pinpoint pupils, albeit with lower frequencies. Psychiatric symptoms were also evident in a subset of cases, albeit less frequently reported. Patients presented with abnormal behavior, incoherent speech, and hallucinations, highlighting the potential for drugs to induce alterations in mood, cognition, and perception. Gastrointestinal symptoms were prevalent among affected individuals, with nausea and vomiting being the most common (40.5%). Additionally, patients reported abdominal pain, diarrhea, gastric discomfort, abdominal distension, and dry mouth. Cardiovascular symptoms, though less common, were observed in several cases, including palpitations, dyspnea, chest tightness, syncope, and chest pain. Respiratory symptoms, such as pneumonia, dyspnea, and wheezing, were reported in a minority of cases. Urinary symptoms, including urinary retention, were observed in isolated instances. Other symptoms reported included fatigue, falls, fever, and blurred vision.

**Table 3:** Clinical symptoms of drug poisoning.

System	Clinical symptoms	Cases/n (%)
Neurological symptoms	Dizziness	43 (51.2%)
	Unsteady gait	25 (29.8%)
	Drowsiness	21 (25.0%)
	Tremor and slurred speech	15 (17.8%) each
	Confusion	7 (8.3%)
	Headache	4 (4.8%)
	Convulsions	3 (3.6%)
	Muscle stiffness and hyperactivity	2 (2.4%) each
	Facial asymmetry, delirium, tinnitus, and pinpoint pupils	1 (1.2%) each
Psychiatric symptoms	Abnormal behavior	4 (4.8%)
	Incoherent speech	3 (3.6%)
	Hallucinations	1 (1.2%)
Gastrointestinal symptoms	Nausea/vomiting	34 (40.5%)
	Abdominal pain	5 (6.0%)
	Diarrhea and gastric discomfort	3 (3.6%) each
	Abdominal distension	2 (2.4%)
	Dry mouth	1 (1.2%)
Cardiovascular symptoms	Palpitations	4 (4.8%)
	Dyspnea	3 (3.6%)
	Chest tightness	2 (2.4%)
	Syncope and chest pain	1 (1.2%) each
Respiratory symptoms	Pneumonia, dyspnea and wheezing	1 (1.2%) each
Urinary symptoms	Urinary retention	1 (1.2%)
Others	Fatigue	34 (40.5%)
	Falls	2 (2.4%)
	Fever	1 (1.2%)
	Blurred vision	4 (4.8%)

## 4 Discussion

This study examined the clinical characteristics of drug overdose in children and adolescents, with a particular focus on gender distribution, age-related trends, and the role of clinical pharmacists in overdose management. Our findings indicated that 83.3% of cases involved females, with a peak incidence at age 15, whereas males exhibited a lower incidence, peaking at age 16. These results are consistent with global trends showing a higher prevalence of medication overdose among adolescent females (5, 18, 19). In addition to gender and age trends, our study identified several important clinical characteristics among adolescents who experienced intentional drug poisoning. Most patients had a diagnosed psychiatric condition, primarily depression, mood disorders, or childhood emotional disorders, and the majority had a history of psychiatric medication use. The most frequently involved drug classes included antidepressants, mood stabilizers, and benzodiazepines, with varying degrees of severity in poisoning outcomes. This pattern aligns with previous observations that psychiatric medication use among adolescents, particularly in China, peaked during the COVID-19 pandemic and has remained elevated through 2023 (20). The pandemic has further intensified suicide risk among adolescents, highlighting the urgent need for targeted mental health interventions (3).

Depression was the most frequently diagnosed psychiatric condition among adolescents who attempted suicide in our study. This is consistent with previous meta-analyses, which have established a strong association between depressive disorders and suicide attempts in adolescent psychiatric populations (21, 22). In our cohort, every patient who experienced drug poisoning had a diagnosis of depression, mood disorder, or a childhood emotional disorder. Despite this elevated risk profile, only a fraction of these patients were flagged as high-risk for suicide at the time of clinical admission (23), highlighting a potential gap in early risk identification or screening processes.

In this study, most adolescents ingested medications that had been prescribed to them. However, several notable exceptions were observed. Two patients without prior psychiatric diagnoses consumed medications prescribed for somatic illnesses, along with the over-the-counter sedative rotundine. One patient ingested a medication intended for an elderly family member with Parkinson’s syndrome, and four others obtained psychiatric drugs, including antidepressants and mood stabilizers, through online sources. These findings reflect increasing concerns about the ease of access to both prescription and non-prescription medications among adolescents and the potential for misuse in terms of suicidal behavior. Previous studies have similarly emphasized the need for stricter monitoring and secure storage of medications in households with at-risk youth (24, 25), as well as the growing public health issue posed by online pharmaceutical access (26).

Moreover, even when psychiatric medications are appropriately prescribed, they can pose risks in vulnerable adolescent populations. In our study, patients treated with antidepressants such as sertraline exhibited a higher lifetime incidence of suicidal behaviors compared to those not receiving pharmacotherapy. These findings are in line with prior research suggesting that selective serotonin reuptake inhibitors (SSRIs) may increase suicide risk in adolescents, particularly during the early stages of treatment (27). Additionally, mood stabilizers such as lithium carbonate and sodium valproate, though effective for managing bipolar and mood disorders, were implicated in overdose events. Notably, while one patient ingested 7.2 g of lithium carbonate, no cases of severe toxicity were observed, which contrasts with earlier reports of significant morbidity from lithium overdose (28). Benzodiazepines, including lorazepam, were another common agent in overdose cases, producing symptoms such as dizziness, ataxia, and coma. Consistent with previous findings (29), we did not observe any instances of severe benzodiazepine toxicity, even at higher doses.

The clinical presentations in drug poisoning cases ranged from mild symptoms such as sedation and nausea to severe toxic effects including ataxia and coma. Several patients required intensive medical interventions, such as hemodialysis, highlighting the potentially life-threatening nature of adolescent overdose incidents and the importance of timely clinical care. Our study found that a significant proportion of drug poisoning patients (34.5%) were taken to the nearest hospital by their families. Due to the patients’ comorbid psychiatric illnesses or psychiatric history, general hospitals often face difficulties in managing such cases, leading to transfers to psychiatric hospitals after initial emergency treatment. Although 94% of patients showed improvement following symptomatic treatment, ongoing specialized psychiatric care was still required to address their underlying conditions. Notably, three patients with severe poisoning required transfer to comprehensive hospitals equipped for blood filtration. These findings align with previous literature reporting that psychiatric medications are not only among the most commonly prescribed drugs for adolescents but are also frequently implicated in intentional overdoses. For instance, a study in Australia found a significant overlap between psychiatric medications commonly prescribed and those used in self-poisoning incidents (30), supporting the observation in our study that the substances ingested were largely psychiatric in nature. This highlights the importance of improved mental health support and oversight in prescription practices for at-risk youth. Similarly, it has been reported that self-harm and suicide attempts among adolescents often involve psychiatric medications initially prescribed for therapeutic use (31), reinforcing the need for close monitoring of this patient population.

Gender, psychiatric conditions, and substance use are well-established risk factors for adolescent overdose (32). In this study, female adolescents were five times more likely than males to engage in overdose behavior. Among all patients, only two had not previously received psychiatric care, despite experiencing prolonged mood disturbances due to physical illness, indicating a high baseline level of mental health concerns across the cohort. The presence of these high-risk factors (being female, having a psychiatric diagnosis, and receiving psychiatric prescriptions), combined with the emotional volatility of adolescence, contributes to impulsive overdose attempts (33). These behaviors not only harm the physical and mental well-being of adolescents but also impose substantial economic and social burdens on families and healthcare systems (34). Consistent with global data (35, 36), our findings reveal gender-specific trends in suicide-related behaviors. While adolescent females more frequently attempt suicide via medication overdose, males are more likely to use more lethal means, resulting in higher suicide mortality rates (33). Regionally, suicide mortality among children and adolescents aged 7-17 in Chongqing increased significantly between 2012 and 2021, with a particularly steep rise in males (APC = 11.85%) (37). These patterns highlight the need for targeted, gender-sensitive interventions.

Clinical pharmacists play vital roles in preventing medication overdose and enhancing the safety of psychiatric pharmacotherapy among children and adolescents. Although pharmacists may not directly manage acute overdose symptoms, their contributions to medication safety and therapeutic optimization are critical. In China, the shortage of psychiatric specialists, with only 1 psychiatrists per 100,000 people compared to a global average of 1.7, and only one-tenth the level seen in developed countries such as Germany, according to the WHO Mental Health Atlas 2020 (38), exacerbates the challenges of mental healthcare delivery. This highlights the need for pharmacists to intervene actively in psychiatric medication management. Pharmacists should provide comprehensive medication counseling when psychiatric drugs are first prescribed, including education on appropriate use, precautions, home management strategies, and the identification and handling of adverse effects. Furthermore, pharmacists can contribute by promoting rational medication practices, such as advocating for limits on single prescription quantities, establishing clear conditions for off-label use, and implementing safety follow-up protocols. In addition to direct patient care, pharmacists at tertiary hospitals can extend their impact by offering training and guidance to primary care institutions on safe psychiatric medication practices. Through interventions at multiple stages of psychiatric care, pharmacists can significantly mitigate the risk of overdose, promote the appropriateness of drug therapy, and improve medication safety outcomes for vulnerable pediatric and adolescent populations.

From a policy perspective, the growing reliance on psychiatric medication among adolescents raises concerns about over-prescription and the need for stricter regulation. The U.S. Food and Drug Administration (FDA) mandates a “black box” warning for antidepressants prescribed to children and adolescents, noting that these medications may increase the risk of suicidal thoughts and behaviors (suicidality) in this age group. Therefore, healthcare providers must carefully weigh the benefits and risks when prescribing these medications, ensuring that they are written for the smallest quantity consistent with effective management. Additionally, patients should be closely monitored for any clinical worsening, suicidality, or unusual behavioral changes, particularly in the early stages of treatment. Families and caregivers are advised to observe patients closely and maintain open communication with prescribing clinicians (39). Moreover, implementing effective strategies for the safe storage and proper disposal of medications at home is crucial, especially in households with at-risk adolescents. The prescription of benzodiazepines and stimulants also carries significant risks. Recent studies highlight the importance of assessing self-injury behaviors in adolescents prescribed these drugs, as they have been implicated in overdose incidents. A study by Bushnell et al. (40) emphasizes the need for targeted risk mitigation efforts in preventing overdoses related to benzodiazepines and stimulants. Given the relatively limited availability of psychiatric hospitals compared to general hospitals, most overdose cases are initially managed at the nearest general medical facility. This highlights the importance of equipping frontline healthcare providers with training in safe prescribing practices and overdose risk mitigation.

This study offers important insights into the prevalence of medication overdose among children and adolescents, informed by the clinical pharmacist’s direct experience in managing this critical issue. It highlights key areas for improvement in pharmaceutical services, including enhanced medication counseling, follow-up, and outpatient care, while providing targeted recommendations for strengthening pharmaceutical management practices, such as addressing off-label drug use, optimizing medication dosages, and managing internet-based prescriptions. Given that the study was conducted at one of China’s largest psychiatric hospitals, whose patient demographics, prescription trends, and suicide attempt rates align with national patterns, these findings are likely to reflect broader trends across similar healthcare settings both in China and globally.

Despite these strengths, several limitations must be acknowledged. First, the relatively small sample size limits the statistical power and generalizability of the findings. Second, the retrospective design of the study may introduce biases related to data collection and analysis. Lastly, the single-center approach constrains the external validity of the results, as they may not fully reflect the broader population or diverse healthcare settings. Future research utilizing larger, multicenter cohorts is warranted to confirm and expand upon these findings.

In conclusion, this study identifies that medications involved in adolescent intentional overdoses are primarily prescribed for therapeutic use, especially psychiatric medications. To mitigate overdose risk, pharmacists should enhance medication counseling during dispensing, clarify treatment expectations, and address misconceptions about psychiatric drug therapy. Additionally, managing access to psychiatric medications and continuously assessing their efficacy and safety is critical. Clinical pharmacists should also be actively involved in the long-term management of psychiatric conditions for ensuring medication adherence and timely interventions. These strategies align with ongoing initiatives such as the Safe Medication Disposal Program and Medication Therapy Management services, which focus on improving medication safety and preventing misuse. Based on our findings, targeted pharmacist-led interventions tailored to high-risk age groups and specific psychiatric conditions may further enhance patient safety and reduce overdose incidents.

## Data Availability

The original contributions presented in the study are included in the article/supplementary material. Further inquiries can be directed to the corresponding author.
